# Breeze 2.0: an interactive web-tool for visual analysis and comparison of drug response data

**DOI:** 10.1093/nar/gkad390

**Published:** 2023-05-13

**Authors:** Swapnil Potdar, Filipp Ianevski, Aleksandr Ianevski, Ziaurrehman Tanoli, Krister Wennerberg, Brinton Seashore-Ludlow, Olli Kallioniemi, Päivi Östling, Tero Aittokallio, Jani Saarela

**Affiliations:** Institute for Molecular Medicine Finland (FIMM), HiLIFE, University of Helsinki, Finland; Institute for Molecular Medicine Finland (FIMM), HiLIFE, University of Helsinki, Finland; Institute for Molecular Medicine Finland (FIMM), HiLIFE, University of Helsinki, Finland; Institute for Molecular Medicine Finland (FIMM), HiLIFE, University of Helsinki, Finland; Biotech Research & Innovation Centre (BRIC), University of Copenhagen, Copenhagen, Denmark; Department of Medical Biochemistry and Biophysics, Chemical Biology Consortium Sweden (CBCS), Science for Life Laboratory, Karolinska Institutet, Stockholm, Sweden; Department of Oncology-Pathology, Science for Life Laboratory, Karolinska Institutet, Stockholm, Sweden; Department of Oncology-Pathology, Science for Life Laboratory, Karolinska Institutet, Stockholm, Sweden; Department of Oncology-Pathology, Science for Life Laboratory, Karolinska Institutet, Stockholm, Sweden; Institute for Molecular Medicine Finland (FIMM), HiLIFE, University of Helsinki, Finland; Department of Cancer Genetics, Institute for Cancer Research, Oslo University Hospital, Norway; Centre for Biostatistics and Epidemiology (OCBE), Faculty of Medicine, University of Oslo, Norway; Institute for Molecular Medicine Finland (FIMM), HiLIFE, University of Helsinki, Finland

## Abstract

Functional precision medicine (fPM) offers an exciting, simplified approach to finding the right applications for existing molecules and enhancing therapeutic potential. Integrative and robust tools ensuring high accuracy and reliability of the results are critical. In response to this need, we previously developed Breeze, a drug screening data analysis pipeline, designed to facilitate quality control, dose-response curve fitting, and data visualization in a user-friendly manner. Here, we describe the latest version of Breeze (release 2.0), which implements an array of advanced data exploration capabilities, providing users with comprehensive post-analysis and interactive visualization options that are essential for minimizing false positive/negative outcomes and ensuring accurate interpretation of drug sensitivity and resistance data. The Breeze 2.0 web-tool also enables integrative analysis and cross-comparison of user-uploaded data with publicly available drug response datasets. The updated version incorporates new drug quantification metrics, supports analysis of both multi-dose and single-dose drug screening data and introduces a redesigned, intuitive user interface. With these enhancements, Breeze 2.0 is anticipated to substantially broaden its potential applications in diverse domains of fPM.

## Graphical Abstract

**Graphical Abstract:**
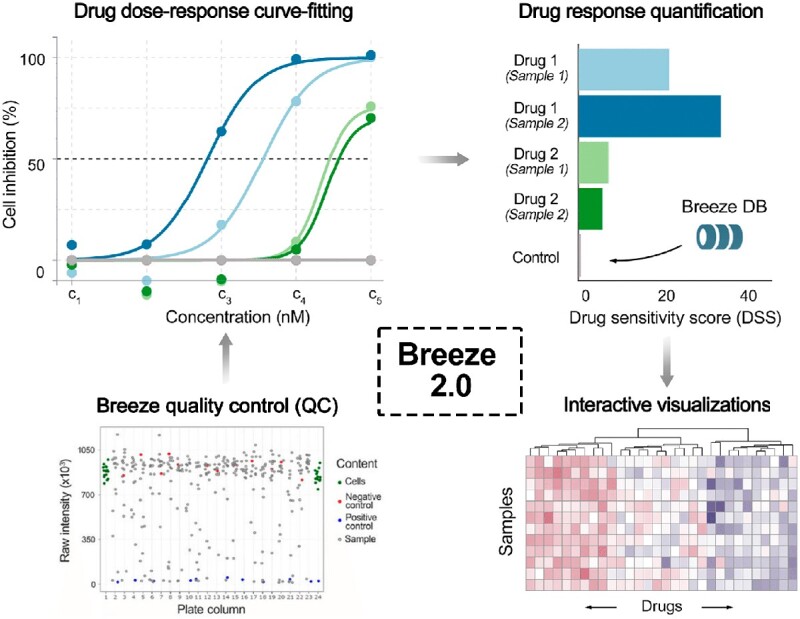

## INTRODUCTION

Development of new drugs and repurposing of existing ones for new indications is a critical and ongoing process with significant potential for enhancing future disease management strategies (1–9). With the escalating prevalence of various diseases and the growing demand for innovative drug development methods, high-throughput screening (HTS) has emerged as a systematic approach for identifying potential hits for drug discovery by profiling thousands of chemical compounds. (10). However, interpreting and analyzing the vast amounts of drug response data generated from the HTS experiments is a complex task, requiring specialized expertise in statistical analysis and programming. Furthermore, the attainment of accurate, interpretable, and reproducible results is essential to identify robust and reliable drug candidates (11,12). One of the key applications of high throughput drug screening lies in the realm of drug repurposing/repositioning. Drug repurposing entails the discovery of novel therapeutic uses for existing drugs, providing a highly effective strategy for the development of drug molecules with innovative therapeutic indications. This process involves the examination of a panel of drugs against specific targets, followed by a systematic comparison with diverse datasets. In response to this challenge, we developed Breeze, a web application for interactive quality control, analysis and visualization of drug dose–response data (13).

Breeze streamlines the analysis and visualization of drug responses generated from cell-based drug screening experiments by implementing comprehensive quality control (QC) procedures, robust dose-response curve-fitting, diverse response quantification metrics, and interactive visualizations. Breeze's QC process plays a crucial role in identifying and quantifying potential errors in data generated from HTS assays, which are prone to common technical issues such as spatial plate variability, striping, and edge effects. Breeze provides a comprehensive set of QC metrics and visualizations, enabling researchers to monitor and identify technical problems, ensuring the accuracy and reproducibility of the screening results. The next critical step involves dose-response curve fitting, which utilizes mathematical modeling to describe the relationship between drug concentrations and the observed responses, such as cell viability or toxicity. Finally, the fitted drug responses are quantified and summarized into single metrics such as half-maximal inhibitory concentration (IC50), half-maximal effective concentration (EC50), area under the curve (AUC), or drug sensitivity score (DSS) to enable comparison across different compounds and concentrations, identifying clinically relevant dose ranges and the most potent and efficient compounds for a given target, patient or disease.

However, Breeze 1.0 did not allow researchers to integrate and compare analyzed datasets with publicly available drug response data, which is crucial for establishing reliable reference baselines for response comparison, validating results, and gaining insights into the broader implications of findings (14). Furthermore, the absence of user-friendly features and automated procedures in Breeze 1.0 posed some challenges for researchers with limited computational expertise. To address these limitations, we have implemented the Breeze 2.0 web-application, which introduces a curated database for easy data integration and comparison, novel interactive visualizations, new drug response metrics, and a redesigned, intuitive user interface. Breeze 2.0 supports analysis of both multi-dose and single-dose drug screening experiments and it utilizes machine learning to flag poor-quality dose-response curves. We believe that the updated web platform will become an even more useful tool, allowing comprehensible and interpretable analysis of drug response data, thus expediting the identification of novel treatment options for various diseases.

## MATERIALS AND METHODS

### Overview of the workflow

Breeze 2.0 introduces a number of novel features and improvements for interactive analysis and visualization of drug response data; these include: (i) a curated database of published drug screening data that facilitates easy integration and cross-comparison of user provided data with the publicly available datasets, including standardized comparison with healthy controls or other reference datasets; (ii) novel interactive visualization options for integrative analysis of user-provided and published data; (iii) implementation of new response metrics for antiviral data analysis; (iv) implementation of a machine learning-based approach for automated identification of poor-quality dose-response curves and (v) analysis of both multi-dose and single-dose drug screening data. Additionally, Breeze 2.0 introduces a re-designed user interface that is more intuitive and user-friendly. Table 1 provides a detailed comparison of the features between Breeze releases 1.0 and 2.0. The users of the Breeze web-application provided valuable input, beta testing and suggestions for improvements which were implemented into Breeze 2.0.

**Table 1.** Comparison of key features between Breeze 1.0 and Breeze 2.0

Feature	Release 1.0	Release 2.0
Comparison with publicly available datasets	No	Yes
Quality control	Plate-based statistics	Plate-based statistics, curve-fitting outlier detection
Minimum number of measured drug doses	4 doses	1 dose
Visualization options	Interactive QC plots, summary barplots and heatmaps	Interactive QC plots, summary barplots, heatmaps, volcano plots, interactive curve fits
Antiviral data analysis	No	Yes

### Data processing pipeline

**Figure 1.**
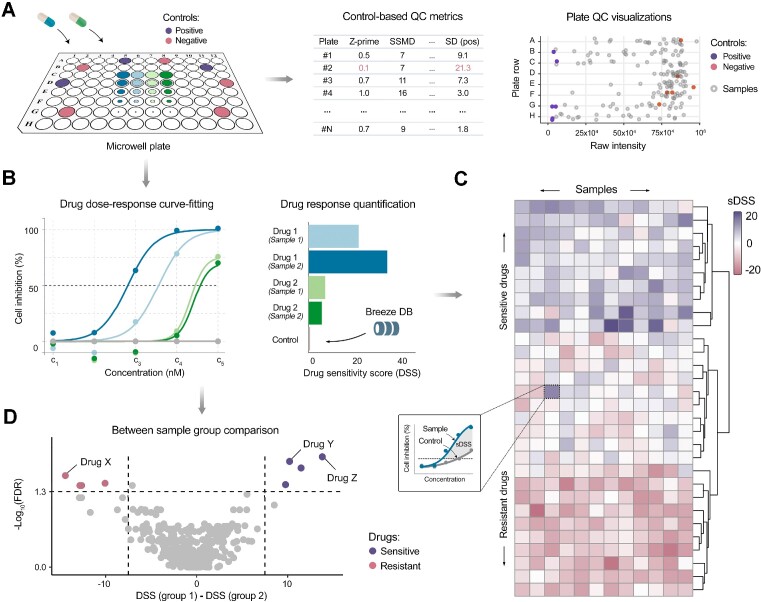
General workflow of Breeze 2.0 web-application. (**A**) Breeze data analysis starts with a quality control (QC) procedure that includes multiple plate control-based QC metrics, such as Z-prime and SSMD (middle panel). Additionally, Breeze features a range of plate-specific visualizations, e.g. scatterplots (right panel), facilitating the detection of anomalies and experimental errors not identified through numerical analysis alone. In this example, Plate #2 demonstrates a poor quality, as evidenced by its low Z-prime score and high standard deviations of positive controls (red highlights in the middle panel). (**B**) Drug dose-response curve fitting is the first step in quantifying drug responses into single metrics (left panel). Subsequently, Breeze 2.0 supports the calculation of various drug performance metrics, including IC50, EC50, AUC and DSS, allowing for direct comparisons between compounds and relative metrics, such as sDSS and SI index that allow for comparison between samples and controls. The barplot illustrates an example where the DSS score was used as the quantification metric (12). Additionally, Breeze offers the possibility to cross-compare user-provided data with previously reported drug responses incorporated into the Breeze database, serving as reference controls for comparison (right panel). (**C**) Next, an interactive heatmap is generated to compare drug responses across different samples (e.g. cell lines or experimental conditions), with sDSS scores shown as an example to highlight the selective efficacy of the drugs, while other metrics can also be used in the heatmap. (**D**) As an alternative to heatmap, statistically significant differences in drug responses between two groups of samples can be identified using a volcano plot.

The Breeze 2.0 pipeline starts with processing of the raw data to generate a comprehensive QC report, featuring plate-specific heatmaps, scatterplots, control barplots, and an in-depth summary of QC statistics emphasizing control well performance. For each drug-dose data point, percent inhibition/viability is determined with reference to the plate controls (Figure 1A). Subsequently, dose-response curve fitting is carried out using four-parameter logistic modeling of percent inhibition values as a function of drug concentration (Figure 1B, left panel).

The curve fitting quality can be visually evaluated using the dose-response curve fitting plots and by analyzing the fitting errors. To ensure improved accuracy, we also employed a machine learning-based model (see the Implementation section) that automatically detects curve fitting errors and flags them in the summary curve fit table. Finally, based on the curve fitting parameters, various drug quantification metrics, such as IC50, EC50, DSS, AUC, are calculated and reported in the summary table (Figure 1B, right panel). In addition, Breeze 2.0 allows calculation of relative metrics, such as selective DSS (sDSS), selective AUC (sAUC) and selectivity index (SI), that enable comparison between samples and controls, and help in the joint assessment of drug efficacy and toxicity. For example, in antiviral drug screening, SI is calculated by dividing a drug's cytotoxicity (its ability to kill cells) by its antiviral activity (its ability to inhibit viral replication), resulting in a ratio that reflects the drug's selectivity for viral targets over host cells. These metrics facilitate the identification of clinically relevant dose ranges and the most potent, effective and selective compounds for specific targets, patients or diseases.

### Breeze 2.0 database

Breeze 2.0 introduces a curated database that allows researchers to integrate and compare their own datasets with publicly available data, which is essential for establishing solid comparison baselines, validating results, and for exploring the wider implications of their findings. The drug responses from the database can serve as a reference control for comparison of user-provided drug responses in the same condition/disease, control groups, or cell lines, identifying disease-specific drug responses and uncovering potent targeted therapies. The database used in the Breeze 2.0 includes data from Malani *et al.* (15), which includes drug sensitivity data from 186 AML patient samples and 17 healthy controls. In addition, we incorporated the PharmacoDB, the most comprehensive database that consolidates pharmacogenomics cell line data from multiple sources such as CCLE, GDSC, NCI-60, CTRP and others (14). In the future, we aim to expand the Breeze database by incorporating additional curated and published datasets, to improve coverage of drug response patterns across a wider range of tissues and drug classes.

### Visualizations

The results of Breeze 2.0 pipeline are visualized in the form of multiple interactive plots such as heatmaps (Figure 1C), volcano plots (Figure 1D), barplots, scatterplots, and circular trees, allowing easy investigation of the results. The details on how to obtain and interpret each visualization plot are explained in the Breeze technical documentation: https://breeze.fimm.fi/DSRT_documentation/docs.html. Within the Breeze interface, users can access the ‘Curve Fitting’ tab and select one or more dose-response curves from a dropdown menu. The software also allows users to incorporate reference data from the database, including information on healthy controls, enabling integrative analysis of drug response data (see e.g. Figure 1B). The resulting plots can be exported as PDF, PNG and HTML files, while a summary table displays drug quantification metrics for selected drugs and screens, which can be downloaded as a spreadsheet.

### Implementation

The Breeze 2.0 web-server is powered by PHP and MySQL for database support. The data processing pipeline utilizes the R programming language and a variety of R packages, while the interactive visualizations are created using GGPlot, Plotly and D3.JS in JavaScript. To ensure the accuracy of curve fitting, an Adaboost machine learning classifier has been trained using Breeze's extensive in-house data set of over 10 000 expert-curated and classified 5-point dose-response curves to flag poor-quality curve-fits in the user data. The final model employs conformal prediction with a 0.8 confidence threshold, which allows users to exclude low-confidence model predictions, thereby flagging only the most confident low-quality curve fits. Currently, the model is capable to flag dose-response curves with a minimum of four doses, up to five drug doses.

## RESULTS AND DISCUSSION

Breeze 2.0 represents a major upgrade to the existing drug screening data analysis pipeline presented in the first version of the tool. Designed to be accessible by researchers with no programming skills, Breeze 2.0 requires only the raw screening data as an input (either raw responses or percentage inhibition/viability), and it automatically analyzes, quantifies and visualizes the drug response data, thereby significantly reducing the manual time required for the analysis of large-scale drug screening experiments.

By incorporating novel data exploration capabilities, users can cross-compare their data in the context of published drug response datasets, enabling the identification of sample-specific, selective drug responses, and avoiding the prioritization of false positive hits, such as generally toxic drugs, when comparing against responses observed in healthy controls. Additionally, Breeze 2.0 enables users to better understand the dose-response relationship with other drugs targeting the same target pathways.

The Breeze 2.0 pipeline's flexible input format and data upload functionality makes it suitable for a wide range of readouts, including numeric data obtained from microscopic images, RNAi experiments, and other sources. This makes Breeze 2.0 a versatile tool that can be used across a variety of different research applications, extending its utility beyond traditional drug development workflows. To the best of our knowledge, there are no similar and equally comprehensive web-platforms for drug response data analysis.

Moreover, Breeze 2.0 features a completely redesigned user interface that is more intuitive and user-friendly. It also includes novel interactive visualization options, which were requested by the users, and essential for avoiding false positive/negative findings. Moving forward, we plan to further expand Breeze by integrating existing and emerging large-scale drug screening resources, with an emphasis on healthy controls from diverse tissues.

## DATA AVAILABILITY

Breeze 2.0 is freely accessible at https://breeze.fimm.fi/v2/ or https://breezetool.app without any login requirements. Extensive documentation of all the features is available at https://breeze.fimm.fi/DSRT_documentation/docs.html.

The Breeze 2.0 database includes published datasets, with links to the original sources provided below. The PharmacoDB datasets were integrated using the PharmacoGx 2.6.0 R/Bioconductor package (https://bioconductor.org/packages/release/bioc/html/PharmacoGx.html), and were downloaded using the downloadPSet function. The Malani *et al.* dataset can be accessed at https://zenodo.org/record/7274740.
